# Attraction of posture and motion-trajectory elements of conspecific biological motion in medaka fish

**DOI:** 10.1038/s41598-018-26186-x

**Published:** 2018-06-05

**Authors:** Atsushi Shibai, Tsunehiro Arimoto, Tsukasa Yoshinaga, Yuta Tsuchizawa, Dashdavaa Khureltulga, Zuben P. Brown, Taishi Kakizuka, Kazufumi Hosoda

**Affiliations:** 1 Graduate School of Information Science and Technology, Osaka University, Yamadaoka 1-5, Suita, Osaka, 565-0871 Japan; 2 Graduate School of Engineering Science, Osaka University, Machikaneyama-cho 1-3, Toyonaka, Osaka, 560-8531 Japan; 3 Graduate School of Frontier Bioscience, Osaka University, Yamadaoka 1-3, Suita, Osaka, 565-0871 Japan; 4 Institute for Academic Initiatives, Osaka University, Yamadaoka 1-5, Suita, Osaka, 565-0871 Japan

## Abstract

Visual recognition of conspecifics is necessary for a wide range of social behaviours in many animals. Medaka (Japanese rice fish), a commonly used model organism, are known to be attracted by the biological motion of conspecifics. However, biological motion is a composite of both body-shape motion and entire-field motion trajectory (i.e., posture or motion-trajectory elements, respectively), and it has not been revealed which element mediates the attractiveness. Here, we show that either posture or motion-trajectory elements alone can attract medaka. We decomposed biological motion of the medaka into the two elements and synthesized visual stimuli that contain both, either, or none of the two elements. We found that medaka were attracted by visual stimuli that contain at least one of the two elements. In the context of other known static visual information regarding the medaka, the potential multiplicity of information regarding conspecific recognition has further accumulated. Our strategy of decomposing biological motion into these partial elements is applicable to other animals, and further studies using this technique will enhance the basic understanding of visual recognition of conspecifics.

## Introduction

Recognition of conspecifics is necessary for a wide range of social behaviours, as it allows effective discrimination among self, kin, predator, prey, competitors, friends, and potential mates^1^; notably, this recognition can be facilitated by various mechanisms, including scent^2,3^, auditory recognition^4–6^, and visual recognition^7^. Visual recognition of conspecifics (VRC), in particular, has attracted research interest and has been observed in a variety of taxa, including both invertebrates^8^ and vertebrates^9^, as well as non-human primates^10^. Given the apparent ubiquity of this phenomenon, understanding the underlying mechanisms in a simple organism would provide useful information applicable in decoding the more complicated processes that are used by other more socially complex systems.

Visual recognition of motion information is one mechanism that can be used to discriminate whether nearby organisms are conspecifics or not; it has been studied using point-light stimuli that replicate the “biological motion” without other associated appearance information, such as colour and detailed shape^11,12^. Multiple taxa of animals can recognise biological motion, including humans^11^, primates^13^, birds^14^, and fish^15,16^. This suggests that motion recognition is important for conspecific recognition and that the neurological mechanism is evolutionarily conserved. Humans have a highly developed ability to recognise biological motion, in which it is possible to identify activity, gender, and emotional state, among other characteristics^17^. Visual recognition of biological motion is impaired in people with social cognition deficits^18^, suggesting that biological motion recognition is related to higher-order social organization. The neurological basis of motion recognition has been investigated^19–21^; however, there are inherent difficulties in analysing the neural system in human studies. Studies involving simpler animals can compensate for those difficulties and can provide information regarding evolutionary aspects.

The shoaling behaviour of fish is a visually striking biological phenomenon that confers various benefits to the shoal members^22,23^ and has provided a useful model system to study VRC^24^. Fish can recognise conspecifics using a variety of parameters, including size^25^, colour^26^, and motion^27^. Additionally, fish possess higher cognitive abilities based on their visual systems^28^, such as discrimination of optical illusion^29^, mirror images^30^, and spatial orientation^31^; these have been established in many neurological investigations^32,33^. Medaka (*Oryzias latipes*), or the Japanese rice fish, is a commonly used model organism^34^ that has been well studied in a variety of contexts (behavioural, neurological, molecular, and systems biology-based) for its comparative cognition^35–38^. Medaka form shoals and schools only with conspecifics and not with other species^39^. Medaka exhibit good eyesight and even possess the ability to recognise faces, enabling identification of conspecific individuals^40^.

In addition to appearance information involving conspecifics, it has been suggested that medaka can recognise motion information alone^15^, as medaka are attracted by biological motion, represented as point-light stimuli that are produced via motion tracking of real medaka. Three-dimensional (3D) computer graphic animations that imitate real medaka have demonstrated that medaka can be attracted by various elements of appearance information, such as colour and shape, and by elements of motion information, such as global motion trajectory and body-shape-level motion of conspecifics; these aspects have been revealed by subtracting each element from the complete 3D medaka animation^41^. These findings suggest that conspecific recognition requires various elements of information, and that medaka can extract information regarding abstract features, even from a portion of the typical elements (*i*.*e*., biological motion alone). However, it is not known whether partial motion elements, such as motion trajectory alone, exhibit attractiveness. Thus, it has not yet been determined what element(s) of biological motion constitute the minimum components of attractiveness.

In this study, to investigate the minimum elements of biological motion for VRC in medaka, we decomposed the biological motion of medaka into either posture or motion-trajectory elements, where the “posture” element contains information regarding body-shape-level motion^42^ (also known as “body motion”)^41^ and the “motion-trajectory” element contains information regarding entire-field-level motion^43,44^ (also known as “locomotion”)^41,45^. We prepared visual stimuli that contain both, either, or none of those elements, using point-light stimuli; then, we presented the stimuli in separate experiments to determine the contribution of each element to the attractiveness of biological motion. We found that each of the two elements alone exhibited a significant degree of attractiveness, which suggested that each element can serve as a minimum component, or that both elements involve a common essential element that has not yet been identified. We also speculated on potential attractiveness characteristics by analysing the stimuli data that we used. To our knowledge, this is the first experiment involving an animal species that used both posture and motion-trajectory elements of biological motion independently. Furthermore, our strategy of decomposing biological motion into these partial elements, and subsequently recombining them, is applicable to other animals. Further studies using this technique will help enhance the basic understanding of VRC.

## Results

### Decomposition of biological motion into posture and motion-trajectory elements

**Figure 1:**
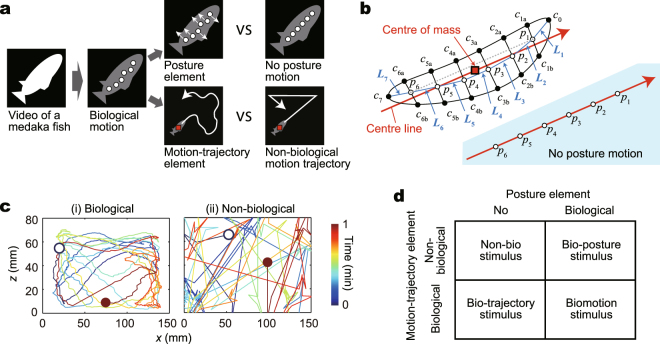
Decomposing biological motion into posture and motion-trajectory elements. (**a**) The conceptual scheme underlying this study. (**b**) Parameters we used for the decomposition. The six points *p*_i_ become the dots of light as stimuli, which are determined from the points on the contour *c*_i_ so that the length of the line segments *L*_1_ to *L*_7_ became nearly equal (see Methods for detail). The angle of the traveling direction was defined as the angle of the line that connected *p*_1_ and *p*_6_ (grey dashed line). The centre of mass was determined directly from the contour, and the centre line was defined as a line that passed the centre of mass with the same angle as the traveling direction. The motion of the centre of mass is defined as motion-trajectory element shown in c. The posture element was defined as the relative motion of each point to the centre of mass in a new rotated coordinate system in which the centre of mass is the origin and the centre line is the horizontal axis. The six dots (*p*_1_ to *p*_6_) in the case of no posture motion are shown at right bottom. In this case, the rotated coordinates of the six dots are kept constant so that the six points are on the centre line with regular intervals. (**c**) The motion-trajectory element of biological and non-biological motion used in this study. The motion in the *x-z* plane for 1 min is shown. The biological motion trajectory was obtained from the motion tracking. The non-biological motion trajectory was designed as linear motion with a constant speed similar to the mean speed of biological motion trajectory. The angle of the turn at the edges was random. (**d**) Four types of stimuli were used.

To develop artificial visual stimuli that are modelled on the biological motion of medaka, we used motion tracking to capture the motion of a single medaka fish and decomposed it into posture and motion-trajectory elements (Fig. 1a). Specifically, we recorded the motion of the individual fish from a side view, and we identified the contour outline of the fish for each time frame. We then converted this contour into six points, such that the six points were set at nearly regular intervals (Fig. 1b; see Methods for details). The six dots correspond to a single medaka fish, and the total length of the six dots approximated that of the actual organism. We used the temporal and spatial changes of the six points as the biological motion stimulus.

To decompose the biological motion into posture and motion-trajectory elements, we determined the “centre of mass of the fish contour” and the “centre line (a line that passed the centre of mass with the same angle as the traveling direction of the motion stimulus)” (Fig. 1b). Information regarding the posture element was extracted from the biological motion as the motion of the six points relative to the centre of mass and the centre line (see legend for detail); then, we compared this biological posture element with “no posture motion,” in which the six points remained aligned in a straight line along the traveling direction of the motion stimulus (Fig. 1b, right bottom). We extracted the motion-trajectory element of biological motion as the motion of the centre of mass, relative to the entire aquarium (Fig. 1c-(i)). We compared this biological motion-trajectory element with non-biological motion trajectory, which is the motion of the centre of mass in a linear fashion with a constant speed that is similar to the mean speed of the biological motion trajectory (Fig. 1c-(ii)), using non-biological motion stimuli as in a previous study^15^. We prepared four kinds of visual stimuli: biomotion (both biological posture and motion-trajectory elements), bio-posture (biological posture element alone), bio-trajectory (biological motion-trajectory element alone), and non-bio stimuli; these incorporated the two types of motions in each posture and motion-trajectory element (Fig. 1d; Supplementary Dataset 2 shows the stimuli video used).

### Observing attraction of medaka by artificial visual stimuli

**Figure 2:**
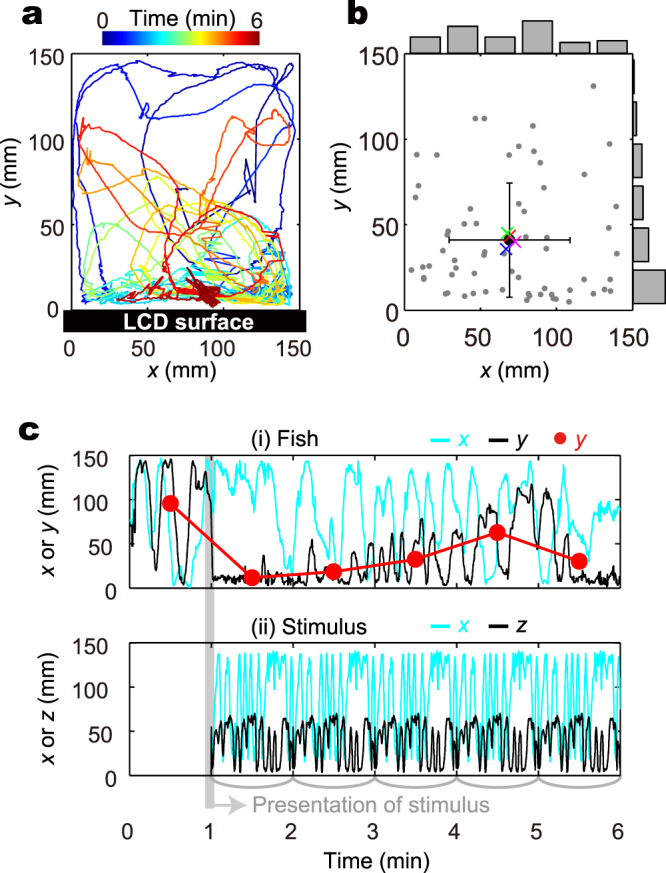
Experimental setup and representative results. (**a**) Observation of a fish from the ceiling surface. LCD was set along with *x*-axis. A representative fish’s motion is shown on the *x-y* axes. (**b**) The mean position of all 64 fish tested. The mean times for 0 to 1 min (with no stimulus presentation) of the *x-y* position of the fish are plotted as grey dots. The mean of all 64 fish is plotted as a black closed circle, with SD for each *x* and y value as the error bar. The mean positions of 16 fish for 4 groups are plotted as coloured crosses. The bars along the outside of the plot box show the histogram of corresponding axes. (**c**) A representative time-series data set of the motion of fish and stimulus. The time course of the *x* and *y* positions of the fish and the time course of the *x* and *z* positions of the stimulus are shown in (i) and (ii), respectively. The red closed circle is the time-mean for 1 min, and the other line shows the data at 30 fps. The stimulus presentation started from 1 min (depicted by grey line). The stimulus was repeated at an interval of 1 min for 5 min in total. There was no interstimulus interval between five loops of the stimulus.

To observe the attraction of medaka by the visual stimuli, we used an LCD display on one side of an aquarium in which a single fish was swimming (Fig. 2a). We recorded the motion of the fish from a location perpendicular to the water surface.

Prior to the initiation of stimulus presentation, we recorded the motion of the fish for 1 min, in order to observe the behaviour of all 64 test fish in the absence of stimuli. Figure 2b shows the time-average *x*-*y* position of each fish for 1 min before stimulus presentation. The *x* position was widely distributed and was almost uniform (Fig. 2b; no significant difference from a uniform distribution in Pearson’s chi-squared test, *χ*^2^ = 5.9, *df* = 5, *P* = 0.06, *α* = 0.05; hereafter, we used the same significance level *α*). The time-averaged *x* was expected to be centred around the aquarium centre if the fish were to swim randomly (*i*.*e*., normal distribution with the mean of 75 mm); however, we observed that some fish tended to prefer the right or left side, even in a symmetric aquarium. Nevertheless, the mean of the time-averaged *x* of 64 fish demonstrated no significant difference with the centre (Student’s *t*-test, *μ*_x_ = 69 mm, *df* = 63, *P* = 0.24); thus, overall symmetry was preserved through averaging of multiple fish. Moreover, there were no significant differences among four groups of 16 fish (coloured crosses in Fig. 2b; ANOVA, *F* = 0.07, *n* = 64, *df* = 3, *P* = 0.98; four groups of 16 fish were presented with one of the artificial stimuli later; additionally, no significant difference in swimming speed was found, Fig. S1). These results suggest that we can statistically represent behaviour characteristics despite large variation among the fish.

Conversely, there was a non-symmetric preference in the *y*-axis direction of the aquarium towards the LCD screen, even prior to presentation of the stimulus (the mean *y* position, *μ*_y_ = 41 mm, was statistically less than half of the aquarium, 75 mm, using Student’s *t*-test, *df* = 63, *P* < 10^−10^; the frequency distribution was significantly different from a normal distribution, as assessed using the Lilliefors test, *P* < 10^−3^). Thus, the LCD screen itself was attractive without stimulus presentation; again, there were no significant differences among the four groups of 16 fish (ANOVA, *F* = 0.24, *n* = 64, *df* = 3, *P* = 0.87). There were some differences between the LCD side and the other three sides. The insides of the other three sides of the aquarium were covered with matte black rubber sheets, while the LCD side remained a shiny glass surface, which can reflect the appearance of the fish, much like a mirror. This reflection might serve as an attractant, similar to that of conspecifics. Moreover, the LCD utilizes a backlight and emits heat, which might also attract the fish.

After the 1 min observation without stimulus presentation, we displayed the visual stimuli for a total of 5 min (five intervals of 1 min video). Figure 2c shows a representative time course of the *x-y* position of a fish and the *x-z* position of an artificial stimulus. We note the sudden attraction of the fish just after the presentation of stimuli, as shown by the rapid decrease in *y* position (distance from the LCD) and gradual relaxation after that (Fig. 2c-(i), black line). The most apparent difference was present between the time-average of *y* at 0–1 min (without stimulus presentation) and the time-average of *y* at 1–2 min (just after stimulus presentation) (red circle in Fig. 2c-(i)). In the following sections, we average the data for each stimulus group of 16 fish and statistically investigate this attraction.

### The attractiveness of the posture or motion-trajectory element alone

**Figure 3:**
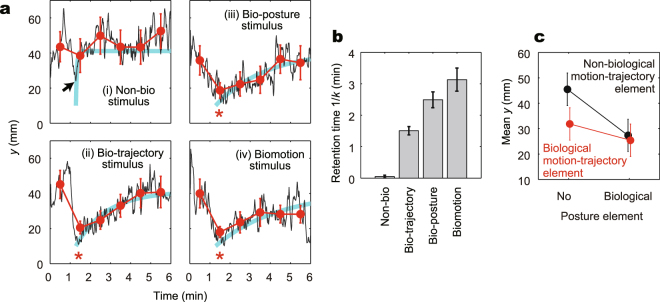
Attractiveness of the 4 types of stimuli. (**a**) Time courses of the position *y* at each type of stimuli. The mean *y* of 16 fish at the time resolution of 1 sec (black lines) and 1 min (for each stimulus intervals; red closed circles with error bars for SEM) are shown. The red stars at 1–2 min show significant decreases from the time-mean of y at 0–1 min by *t*-test, and the black arrow indicates the time point whose value shows significant decreases from the time-mean of *y* at 0–1 min in *z*-test (see text for detail). The cyan curves show the fitting of the data to a single exponential relaxation curve *y* = (*y*_0_ − *y*_eq_) ∙ [1 − exp(−*k*(*t* − *t*_0_))] + *y*_eq_, where *t*_0_ is the start of relaxation determined as the most attracted time, *y*_0_ is *y* value at *t*_0_, and *y*_eq_ is *y* value at equilibrium (we used the common values for all 4 fittings: *t*_0_ = 77 sec and *y*_0_ = 10 mm as the minimum value of all 4 stimuli, and *y*_eq_ = 41 mm as the mean *y* for 0–1 min without stimulus presentation). We also calculated the time courses of the position *y* normalized by the *y* value at no stimulus presentation of each fish, and we found not much differences (Fig. S2b). (**b**) The fitting results of the retention time 1/*k* of the relaxation curves. The error bars show the 95% confidence intervals of the estimates with the Bonferroni correction for the _4_C_2_ of pairwise comparison. (**c**) Interaction plot of multi-factor ANOVA for the *y*-position (see text for detail). The error bars show 95% confidence intervals with Tukey’s HSD.

To determine whether the posture or motion-trajectory element alone is attractive, we analysed the time course of changes in the *y* position of the fish during presentation of each stimulus. We averaged the changes in the *y* position of 16 fish for each stimulus (Fig. 3a) to statistically obtain the specific feature of attraction from within the large variation among fish, as above (Fig. S2a for all data before averaging). There was a clear attractive process, except when fish were presented with the non-bio stimulus. More specifically, the time-mean of *y* at 1–2 min (just after stimulus presentation) was significantly less than the time-mean of *y* at 0–1 min (without stimulus presentation) for biomotion, bio-posture, and bio-trajectory stimuli, but not for the non-bio stimulus (Fig. 3a, red circles; Welch’s *t*-test with the Benjamini-Hochberg procedure at *FDR* < 0.1; *df* = 65, 44, 55, and 21, and *FDR* = 0.0002, 0.001, 0.001, and 0.81, respectively). Thus, each of the two elements was attractive.

Although the non-bio stimulus exhibited no significant attractiveness in the minute-averaged data, the minimum *y* value of 1 sec resolution was observed just after the stimulus presentation (black line in Fig. 3a-(i); *y* = 24 mm at 17 sec after 1 min, black arrow), which was significantly different from the time-mean of *y* for 0–1 min without stimulus presentation (*z*-test; *μ*_y_ = 41 mm, *σ* = 8.5 mm, *P* = 0.023). This rapid attraction suggests that once the fish approaches and can assess the non-biological elements, it is able to determine that the stimulus is not a conspecific and will then ignore the stimulus. Indeed, our analyses using the convergent cross mapping^46^ suggested that their behaviour somehow depended on the motion of the stimuli in a short timescale of <1 sec (Fig. S3), even in the case of a non-bio stimulus. Hence, the fish were initially attracted by the stimuli and then relaxed; the differences across four stimuli appeared to arise from the rate of relaxation.

To quantitatively evaluate the attractiveness, we fitted the time course to a single exponential relaxation curve. We used one fitting parameter *k*, the relaxation rate, to simply express the time courses and compare them with one representative index (Fig. 3a, blue lines). Figure 3b shows the fitting results of the retention timescale 1/*k*. We found that each of the posture and motion-trajectory elements exhibited significant attractiveness, and that the contribution of the posture element was slightly greater than that of the motion-trajectory element. Additionally, we found that the non-bio stimulus exhibited very little attractiveness, although the non-bio stimulus contained information regarding the size and swimming speed of the fish.

We then investigated the interaction of the two factors of posture and motion-trajectory elements by multi-factor ANOVA for the *y* position with three different factors: posture element (two types: biological or non-biological), motion-trajectory element (two types: biological or non-biological), and time (five points). Figure 3c shows the interaction plot, focusing on the two elements. Both posture and motion-trajectory elements had significant effects (*F* = 12, *df* = 1, *P* < 10^−3^ and *F* = 4.9, *df* = 1, *P* = 0.03, respectively); we did not detect a significant interaction effect between the two elements (*F* = 2.8, *df* = 1, *P* = 0.1).

### Quantitative analyses of the stimuli data

Our results above show that both posture and motion-trajectory elements separately demonstrated a significant contribution to attraction in medaka. Here, we quantitatively analyse the stimuli data to determine what stimuli information the fish used to distinguish between biological and non-biological motion.

**Figure 4:**
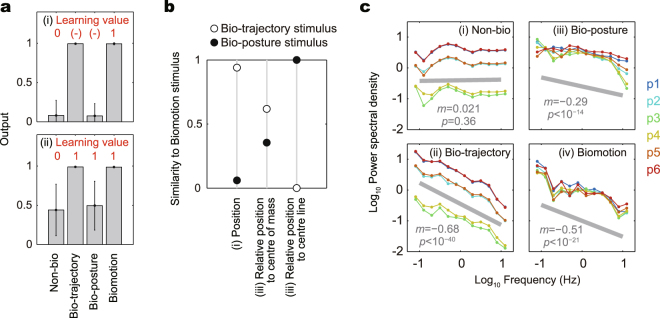
Analyses of stimuli data. (**a**) Recognition of the stimuli data by ANN. After the training process of the stimuli data using the indicated learning values, we input 100 data with random initial time for each of four stimuli to the trained ANN. The bars and error bars show the mean and SD of the output value of 100 data for each of the four stimuli. (**b**) Similarity of the stimuli. The relative similarity of bio-posture and bio-trajectory stimuli are shown. The relative similarity was calculated as 1 − *d*_B_/(*d*_B_ + *d*_N_), where *d*_B_ and *d*_N_ are the Euclidean distance of the stimulus from biomotion and non-bio stimuli, respectively. Simply, the relative similarity becomes 1 and 0 if the stimulus is identical to biomotion and non-bio stimuli, respectively. (**c**) PSD of the four stimuli. We calculated the speed of the change in the relative *x-z* position to the centre of mass for each of six points, and we obtained the PSD of the time-series speed data by fast Fourier transform. We calculated the logarithmic mean of PSD at each range of frequency with logarithmic bins, which are plotted as dots. To determine the slope, we normalized PSD by its mean value for each of six points and linearly fitted the all data for each stimulus. The slope and its *p*-value for the correlation are indicated in the graph, and the grey lines show the slope at an arbitrary intercept.

First, to roughly assess how simple the observed distinction ability is, we compared it with pattern recognition employed by a simple artificial neural network (ANN), which can only solve low-order tasks, such as linear problems. Note that ANN here serves solely as an example of a simple recognition function; there is no relation between the ANN and the fish’s neural network in either structure or performance. Nevertheless, the comparison with an artificial system can provide an indicator of the function of a biological system. We assumed the fish had previously observed biomotion and non-bio stimuli and that they observed bio-posture and bio-trajectory stimuli for the first time. To simulate the situation, we trained a simple single-hidden-layered ANN using time-series data, including the *x-z* position of biomotion as 1 and non-bio stimuli as 0. We then input all four stimuli into the trained ANN. We found that the simple ANN identified a bio-trajectory stimulus as biomotion stimuli but identified a bio-posture stimulus as a non-bio stimulus (Fig. 4a-(i)), which differed from the experimental results. Even when we trained the ANN by the use of all four stimuli, the simple ANN could not correctly identify the difference within the posture element (Fig. 4a-(ii)). This result is reasonable because the difference in the posture element is much smaller than the difference in the motion-trajectory element in terms of the scale (*i*.*e*., in Euclidean distance of the time-series position data) (Fig. 4b-(i)). Our analyses show that, whereas it is difficult for a simple artificial recognition function to distinguish the difference in posture element, it is possible for the fish, which suggests that the fish use a high-order strategy, such as normalizing the motion-trajectory element by focusing on the object.

To elucidate how medaka can distinguish a bio-posture stimulus from a non-bio stimulus, we quantified some information regarding our stimuli. We checked whether the body-shape-level position of the six points, relative to the centre of mass (without normalizing the traveling direction of the stimulus), was similar between biomotion and bio-posture stimuli. We found that the bio-posture stimulus remained more similar to the non-bio stimulus than to the biomotion stimulus (Fig. 4b-(ii)) because the changes in the traveling direction were still dominant. As a definition of the bio-posture stimulus in this study, bio-posture and biomotion stimuli are identical in regard to the position of the six points relative to the centre of mass and the centre line (*i*.*e*., information regarding traveling direction was added to the relative position) (Fig. 4b-(iii)). Therefore, recognising traveling direction might be one of the keys to distinguishing differences in the posture elements, although this remains speculative.

To identify potential information other than the traveling direction, especially regarding the parts-level for each dot motion, we investigated the power spectrum densities (PSD) of each dot motion. PSD is the distribution of the intensity in the frequency spectrum; a power-law PSD is known to induce a predation behaviour (but not attraction) in the case of a single dot that imitates plankton^47^. Figure 4c shows the PSD of the speed of change in the position of the six points, relative to the centre of mass. We found that the three biological stimuli (biomotion, bio-posture, and bio-trajectory stimuli) followed a power law with significant negative slopes; only the non-bio stimulus did not follow the power law. Thus, if the fish can extract information regarding the power law fluctuation, they can distinguish the three biological stimuli from the non-bio stimulus, regardless of the distinction between posture and motion-trajectory elements. This power law fluctuation might be an essential element for the attraction. As above, we speculated on the information that was used to distinguish differences in posture elements, such as the relative position, traveling direction, and the power-law fluctuation.

## Discussion

We decomposed the biological motion of live medaka into posture and motion-trajectory elements and found that each element exhibited significant effects on attraction without significant interaction between the elements; this suggests that medaka can rely separately on information in both of these partial motion elements to identify conspecifics. Our finding of being sensitive to both elements of biological motion information separately would be beneficial for the survival of medaka, as they could find both near and far distanced conspecifics. This logical disjunction (OR) operation of the two elements is suitable to avoid overlooking conspecifics, while the logical conjunction (AND) operation is advantageous for ignoring unclear information; the use of the OR-type operation seems to be reasonable because overlooking conspecifics would be fatal for the social fish. To achieve effective social behaviour, it is important to combine multiple visual features, which are detected as separated and specialized features (*e*.*g*., colour, shape, and motion in the earlier visual processes)^48^, into unified higher representations of the surrounding environment^49^. Although these binding processes of visual stimuli are thought to be challenging tasks^49–51^, Neri *et al*. showed that zebrafish can combine multiple different visual stimuli (morphology and motion)^52^. Our results further suggest that it is possible for a fish to combine detailed multiple partial elements of motion information separately.

Interestingly, our results of separable effect by the multiple elements of biological motion is consistent with the neurological knowledge of human visual recognition. The point-light stimulus can provide well-refined motion information without other appearance elements of conspecific visual information. Nevertheless, the human neural system is considered to further decompose point-light stimulus by two separate pathways for “form” and “motion”^53^. It has been experimentally shown that form and motion are recognized by different area of the human brain^54–57^, *e*.*g*., the extrastriate and fusiform body areas (EBA and FBA) and the inferior temporal sulcus (ITS) for form, and the posterior superior temporal sulcus (pSTS) and ventral premotor cortex (vPMC) for motion. Similar results were also obtained in monkeys^58^. Our results agree with such separate mechanisms for perceiving biological motion by showing the separate contributions of the two elements, although the two elements in this study are different from the form and motion information discussed above. Specifically, the posture element in this study includes both the form and motion information, and thus our speculation of the higher-order strategy for the posture element would suggest the existence of a mechanism of further decomposition of the posture element. Thus, at least three parts of the brain (one for the motion-trajectory element and two for the posture element) might be sensitive to point-light biological motion in medaka. As above, our study added the suggestion of the separate mechanism for perceiving biological motion also in fish, which is important for considering future neurological studies.

Given the comparative neurological simplicity of medaka as a model organism, further analyses of neurological approaches are required for a more general understanding of neural systems. Fish and human brain have similarities in the main structure: both have a cerebellum, midbrain, thalamus, and cerebrum (telencephalon)^59–61^. One of the major difference is the highly-developed cerebral cortex of humans, which includes the visual cortex necessary for visual processing of biological motion. Perceiving biological motion is also accompanied by activation of the premotor cortex^62^, known as a type of mirror neurons^63^, which fire both at their own action and upon observation of the same action in another conspecific individual. Thus, most of the parts related to perceiving biological motion belong to the cerebral cortex. On the other hand, the fish cerebrum is considered to play a much less role than the human cerebrum, and the main parts for visual processing and locomotion are considered to belong to the midbrain^64^ and hindbrain^65^, respectively. Nevertheless, our study and previous studies^15^ suggest that fish can recognise conspecific biological motion, which could be explained by either (i) previously unrevealed functions of the fish cerebrum, or (ii) usage of other brain regions than the cerebrum, *i*.*e*., the cerebrum evolved (from fish to human) to strongly support or replace many functions of other brain regions such as midbrain and hindbrain. Both hypotheses could be true, *e*.*g*., (i) new functions of the fish cerebrum has been revealed by contemporary sophisticated studies^66,67^, and (ii) a recent study of the human brain showed that a presumably motoric thalamic area was sensitive to biological motion^68^. The thalamus is considered to be a more primitive region than the cerebral cortex, and the study suggested the origin of perceiving biological motion comes from a primitive subcortical network, which might share some similarities with the fish brain. Therefore a common fundamental basis may exist, based on the separate mechanisms outlined above. The separate mechanisms seem to be conserved and the next challenging step is to understand the dynamics of the whole neural system, where the simpler fish brain may offer advantages to finding the common fundamental basis. Therefore, revealing the neurological basis of how the fish brain, with its simpler structure, manages to recognize the conspecific biological motion will strongly contribute to both the understanding of the fundamental mechanisms of complex brain tasks and their evolutionary origins.

Further quantitative study is required to investigate what extent medaka recognise conspecifics from the biological motion. It is known that human biological motion stimuli can attract medaka, although the attractiveness is less than that of medaka biological motion stimuli^15^. Additionally, we found that the difference in attractiveness between the biological and non-biological motion was quantitative, rather than qualitative (Fig. 3). Therefore, for understanding the extent of conspecific recognition from motion information alone, it is required to quantitatively compare the attraction between conspecific biological motion stimuli and many other stimuli, which include biological motion stimuli from various species, as well as various artificial stimuli. Such an experiment is possible using our quantitative method (as shown in Fig. 3b) and will also highlight the elements of motion information used for conspecific recognition, from similarities and differences of the stimuli.

Our analyses used various basic methods that might be useful for future studies. For instance, to quantify the attractiveness, we applied the time course of simple mean distance, whereas previous studies have applied more sophisticated methods using *a priori* knowledge, such as binary evaluation of attraction with a specific threshold and exclusion of fishes in a certain condition^15,41^. Our basic methods, which do not incorporate *a priori* knowledge and normalization, may be useful for future data-driven approaches such as in other species because such basic methods are robust, although the quality of the data analysis might be slightly lower than in sophisticated analyses. Also, we used convergent cross mapping^46^ to check whether the fish behaviour somehow depended on the stimuli motion in a short timescale (Fig. S3). The convergent cross mapping has been usually used in the field of ecology and earth science, but our study suggested that it would be also useful for investigating animal behaviour. Moreover, we used a simple ANN to characterize potential features of the fish’s recognition. There is no relation between the ANN and the fish’s neural network in terms of either structure or performance; however, ANN may be useful as a reference indicator to highlight features regarding the function of biological systems, *e*.*g*., a more complex ANN (or a more layered ANN) can solve more complex problems. Because we can prepare various ANNs that differentiate quantitative and qualitative aspects, these can be used as indicators of the function of biological systems.

Our reconstructive approach, employing the decomposition of biological motion into posture and motion-trajectory elements, is applicable to the induction of social behaviours in other animals, including humans, where biological motion has been decomposed into “style” and “timing,” then recombined to design artificial human motion^69^. Such technology regarding motion synthesis and a related database is useful to investigate the effects of various partial elements of biological motion. Moreover, in the case of social interactions that include multiple individuals simultaneously, posture and motion-trajectory elements may exhibit more complex relationships. Further approaches that expand our reconstructive approaches into group interactions^70,71^ are necessary, as they would elucidate the connection between the visual recognition of conspecifics and more complex social interactions.

## Methods

### Fish and housing conditions

Adult medaka (Oryzias latipes, orange variety) were purchased locally (Petland Gorilla, Minoo-imamiya, Japan) and kept in 13-L grass tanks containing no more than 20 individuals for at least 7 days prior to testing. Treated municipal water (Killifish Water, Spectrum Brands Japan, Japan) was used for housing and was kept aerated and filtered at 26 ± 1 °C. Fish were fed a dry diet (Kyouzai medaka no esa, Kyorin, Japan) at 9:00 and 18:00 each day. The housing tanks were lit starting at 8:00 for 14 hours, and all of the experimental manipulations were conducted between 9:00 and 17:00. All animal care and experimental procedures conformed to the guidelines established by the Osaka University Animal Experimentation Committee.

### Motion tracking of fish

Motion tracking of medaka fish was conducted to prepare biological motion stimuli. A glass cubic aquarium (15 × 15 × 15 cm, lwh) containing a depth of 8.0 cm of housing water (24–26 °C) was used as a filming tank, with the bottom and three side walls of the aquarium covered with black rubber sheets to restrict external stimuli and the remaining wall left transparent. A single fish was transferred to the aquarium for 23-hour habitation^72^ before moving the aquarium in front of a digital video camera (Firefly MV 1394a, Point Grey, Canada) with fluorescent lighting adjusted to 500 lux brightness at the water surface. After 50 minutes of habitation, 5 minutes of video were recorded at 30 fps. The stimuli were generated using 2 to 3 min of the video.

For each frame, the contour of the fish was detected using C++ and OpenCV (Open Source Computer Vision; http://opencv.org/). We converted the contour into six points by maximizing the following fitness function:

1 $$fitness=\frac{{\sum }_{i=1}^{7}{L}_{i}}{\sqrt{\{4({{L}_{1}}^{2}+{{L}_{7}}^{2})+{\sum }_{i=2}^{6}{{L}_{i}}^{2}\}-{\{2({L}_{1}+{L}_{7})+{\sum }_{i=2}^{6}{L}_{i}\}}^{2}}}$$

Here, *L*_1_ to *L*_7_ are the lengths of the seven links (see Fig. 1b) between eight points (*c*_0_, *p*_1_ to *p*_6_ and *c*_7_). *p*_*n*_ (*n* = 1, …, 6) are the middle points of *c*_*na*_ and *c*_*nb*_ respectively. Initially, the fourteen points (*c*_0_, *c*_1*a*_ to *c*_6*a*_, *c*_1*b*_ to *c*_6*b*_ and *c*_7_) were located on the contour at even intervals. Then, the fitness function was maximized by iteratively shifting the points in a random direction 1000 times so that *L*_1_ to *L*_7_ became nearly equal. In this study, we regarded time changing of *p*_1_ to *p*_6_ (white circles in Fig. 1b) as biological motion information, which was extracted by converting the contour of the fish.

### Stimulus presentation to fish

Cubic aquaria and fish were prepared as described above. The test aquarium was placed in front of an LCD display with a black screen, and 50 minutes was given for habituation; then, one of the artificial stimuli was presented for 5 minutes. The behavior of each fish was recorded with a digital camera (Firefly MV 1394a) from above the test aquarium for 6 minutes to include 1 minute prior to stimulus presentation and the video was quantified by almost same program used for the motion tracking above. The contour of the fish was directly converted to its central coordinate and evaluated. Each fish was exposed to only one of four kinds of stimuli, in order to avoid the effect of hysteresis of other stimuli.

### Convergent cross mapping

We used R software^73^ and its package rEDM. We used the time-series data at 30 fps of tested fish position and stimulus position for 3 min from just after the start of visual stimuli presentation. We applied the data to the function “ccm” with the embedding dimension 3.

### Analyses of the stimuli by ANN

For the ANN analyses, we used MATLAB R2014a (MathWorks, MA, USA) and the function “patternnet” to set a simple single-hidden-layered ANN at the size 10. We used the time-series data of *x-z* position of the stimuli for 1 min at 30 fps (1800 frames). We randomly selected 100 frame points for the starting point (time frames were treated as circular structure) for training data. We trained the ANN by using the 100 time-series stimulus data for each stimulus with target values of 1 or 0. We then prepared another randomly selected 100 time-series for each of four stimuli as input data and obtained output data.

### Data Availability

All data generated or analyzed during this study are included in this published article (and its Supplementary Information files).

## Electronic supplementary material

Supplementary information

Dataset 1

Dataset 2

Dataset 3
