# Supplementary material for: Attraction of posture and motion-trajectory elements of conspecific biological motion in medaka fish
Source: Sci Rep. 2018 Jun 5;8:8589. doi: 10.1038/s41598-018-26186-x (PMC5988670; doi:10.1038/s41598-018-26186-x)
Supplement: Supplementary file 1 — Supplementary information [file 41598_2018_26186_MOESM1_ESM.pdf]

Supplementary information

Attraction of posture and motion-trajectory elements of conspecific biological motion in medaka fish

Atsushi Shibai, Tsunehiro Arimoto, Tsukasa Yoshinaga, Yuta Tsuchizawa, Dashdavaa Khureltulga, Zuben P. Brown, Taishi Kakizuka, and Kazufumi Hosoda

This file includes figures S1, S2, and S3 (see below).
Datasets 1, 2, and 3 are uploaded as separate files.

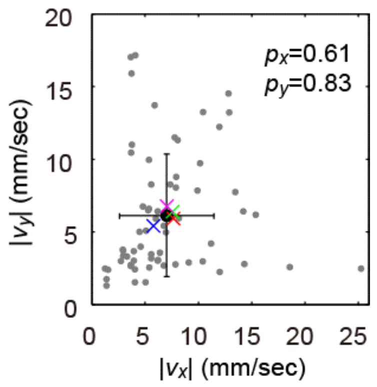

Figure S1. The mean speed of all 64 fish tested. The time mean for 0 to 1 min (while no stimulus presentation) of speed in x and y axes ($|v_x|$ and $|v_y|$, respectively) of the fish are plotted as gray dots. The mean of all 64 fish is plotted as a black closed circle, with SD for each $|v_x|$ and $|v_y|$ values as the error bar. The mean speed of 16 fish for 4 groups are plotted as colored crosses, and the p-values in ANOVA are indicated (there were no significant differences between 4 groups of 16 fish).

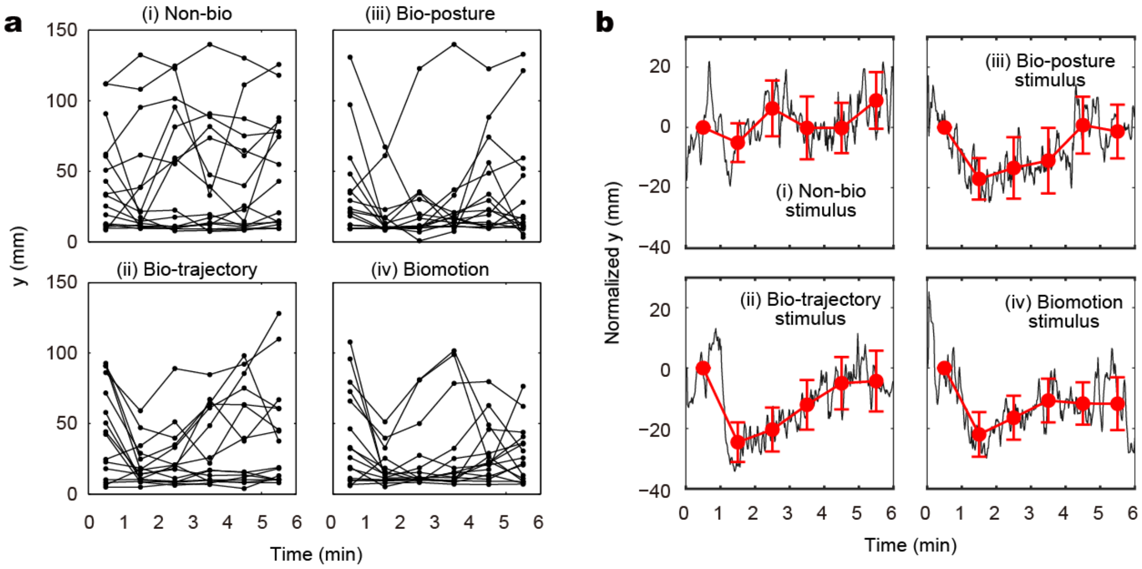

Figure S2. Time courses of the attraction under the presentation of each type of stimuli. (a) Time courses of the position *y* for all 64 fish. The time course of 16 fish for each stimulus are shown. The mean value of these 16 fish are shown in Fig. 3a in the main text. (b) Time courses of the position *y* normalized by the *y* value at no stimulus presentation. We obtained the time series of normalized *y* by subtracting the time mean of *y* for 0 to 1 min (with no stimulus presentation) from the time series of *y* for each fish. The data is presented as the same as Fig. 3a.

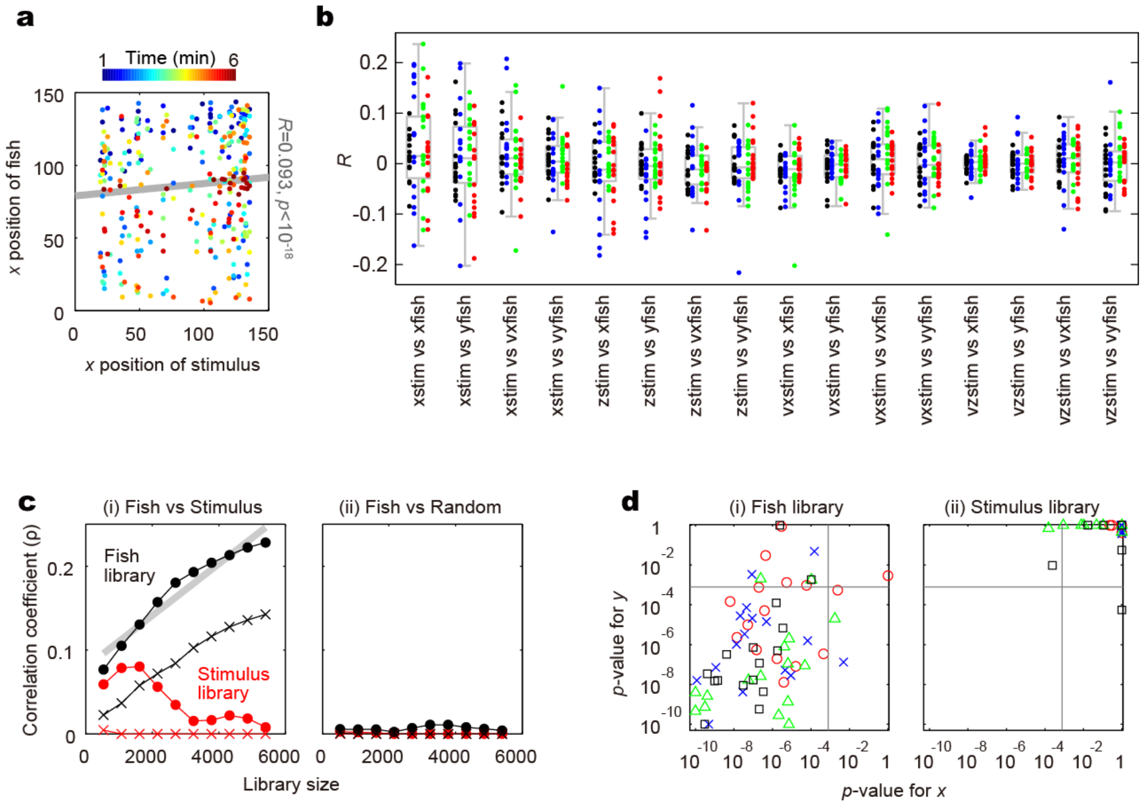

Figure S3. The effect of the visual stimuli on the behavior in a shorter timescale. The analysis we conducted in the main text was based on a minute-average scale, but organism activity occurs on a shorter timescale. If medaka are responding to stimuli over short time periods, then they may change their behavior quickly depending on the presented stimuli. To investigate this, we extracted information about quicker responses to the visual stimuli from the 30 fps data we collected. We investigated various correlations between a fish's motion and a stimulus's motion (a and b), and dynamic causal relationships between fish motion and stimulus motion (c and d). We did not find any strong correlations, but the causal analysis suggested that the fish were watching the stimuli even at a non-bio stimulus and their behavior somehow depended on the stimuli's motion in the short timescale, as follows.

Initially, we investigated various correlations between a fish's motion (position or velocity on *x*- and y-axes) and a stimulus's motion (position or velocity on *x*- and z-axes). (a) The correlation between *x* position of stimulus and *x* position of a fish as a representative correlation. The correlation coefficient and its *p*-value of 30 fps data are indicated (the plot shows its time-average for 1 sec because 30 fps data is too many). The

correlation was significant due to the large data size, but it was too weak to extract information. (b) Various correlations. The correlation coefficient between fish's behavior (position on *x* and *y* axes, shown as xfish and yfish, respectively, and speed on *x* and *y* axes, shown as vxfish and vyfish, respectively) and stimuli's variables (position on *x* and *z* axes, shown as xstim and zstim, respectively, and speed on *x* and *z* axes, shown as vxstim and vzstim, respectively) are shown. Each dot shows a correlation about a single fish experiment. Red, green, blue, and black dots show the results using biomotion, bio-posture, bio-trajectory, and non-bio stimuli, and the gray box plots of all 64 fish for each correlation are overlaid. There were many significant correlations just like the representative above, but there was no mean correlation coefficient of 16 or 64 fish that was significantly different from zero in *t*-test with the Benjamini-Hochberg procedure at $FDR<0.1$, *i.e.*, there was no significant dependences.

Then we investigated dynamic causal relationships between fish motion and stimulus motion. Obviously, this causal relationship is one-directional, as the stimulus motion can be causal with the fish's motion, but the opposite can never occur. If medaka are responding quickly to a stimulus and behave quickly depending on the stimulus's behavior, we can detect the stimulus's motion as a cause of a fish's motion without detection of the opposite causality. We used the convergent cross mapping (CCM) to detect the causality. When we consider a unidirectional causality in which a stimulus's *x* position (xstim) causes a fish's *x* position (xfish), the xfish time series includes the information of both xstim and xfish because xfish dynamics is determined by both, while the xstim time series does not include any information about xfish. Therefore, xstim can be reconstructed in a certain quality from the data library of the xfish time series. In this reconstruction, the quality of the reconstruction (correlation between the reconstructed xstim and the actual data of xstim) should continually improve, as longer time series (larger library size of data) are used for reconstruction, but the quality should not increase in the opposite case of reconstruction of xfish by xstim. Note that the direction of reconstruction (xfish data library to xstim) is opposite to the causal direction (xstim to xfish).

The results are as follows. (c) Results of CCM for the relationship between fish's motion and stimulus's motion (i) and between fish's motion and a random time-series data as a negative control (ii). The circles indicate the relationship between the fish's *x* position and the stimulus's *x* position, and the crosses indicate that between the fish's *y* position and the stimulus's *x* position. The black plots indicate the correlation coefficient between the stimulus data and the reconstructed stimulus values by using the library of fish's time-series data, and the red plots indicate the opposite. The gray line shows a representative linear regression whose *p*-value of correlation with a positive slope is shown in d. In (i), the quality

of reconstruction of xstim was continually improved with increasing library size of xfish data (black circle; with a significant positive slope of 3.1×10^{-5} with $P<10^{-5}$, gray line) but not vice versa (red circle). We obtained similar results in the case of a fish’s *y* position (black and red cross). These results suggested that the fish behavior depended on the motion of the stimuli over a short timescale (less than 1 sec), not only in the *y*-axis direction (attraction) but also in the *x*-axis direction, although it was difficult to explore the detail of the dependency, as the correlation of reconstruction was still low. We checked that these results were not observed when we conducted CCM by virtually replacing xstim with random data (ii). The significance of the slope for all samples are shown in d. (d) The *p*-value of correlation with a positive slope between stimulus data and reconstructed stimulus values by using the fish's data library (i) and the opposite (ii). The horizontal axis indicates the results for the relationship between the fish's *x* and the stimulus’s *x*, and the vertical axis indicates that between the fish's *y* and the stimulus's *x*. Red circles, green triangles, blue crosses, and black squares indicate the results of experiments using biomotion, bio-posture, bio-trajectory, and non-bio stimuli, respectively. Samples with a negative slope are plotted at 1, and samples whose values under 10^{-10} are plotted at 10^{-10}. The gray lines show the significant level with the Bonferroni correction. A significant slope (*i.e.*, causality) was detected in all experiments except one, for the case assuming a stimulus’s motion causes fish’s motion, at least in either the *x* position or the *y* position (i). Thus, even a non-bio stimulus had some causal relationship with the fish’s motion. In contrast, all experiments except for three showed no significant slope in both the *x* position and the *y* position for the case of the opposite directional causality (ii).
